# Inherited burden for disease predisposition in diverse populations

**DOI:** 10.1038/s41525-026-00552-5

**Published:** 2026-02-18

**Authors:** Barış Kayaalp, Meltem Ece Kars, Yuval Itan, Ayşe Nazlı Başak, Jean-Laurent Casanova, Tayfun Özçelik

**Affiliations:** 1 Department of Molecular Biology and Genetics, Faculty of Science, Bilkent University, Ankara, Türkiye; 2 Charles Bronfman Institute for Personalized Medicine, Icahn School of Medicine at Mount Sinai, New York, NY USA; 3 Department of Genetics and Genomic Sciences, Icahn School of Medicine at Mount Sinai, New York, NY USA; 4 Mindich Child Health and Development Institute, Icahn School of Medicine at Mount Sinai, New York, NY USA; 5 Suna and Inan Kıraç Foundation, Neurodegeneration Research Laboratory, Research Center for Translational Medicine, Koç University School of Medicine, Istanbul, Türkiye; 6 St. Giles Laboratory of Human Genetics of Infectious Diseases, Rockefeller Branch, Rockefeller University, New York, NY USA; 7 Laboratory of Human Genetics of Infectious Diseases, Necker Branch INSERM U1163, Necker Hospital for Sick Children, Paris, France; 8 Imagine Institute, University of Paris, Paris, France; 9 Pediatric Immunology-Hematology Unit, Necker Hospital for Sick Children, Paris, France; 10 HHMI, New York, NY USA

**Keywords:** Genetic variation, Medical genomics, Genetic predisposition to disease

## Abstract

We leveraged allele frequencies from gnomAD, Regeneron Genetics Center Million Exome and Turkish Variome for 4591 disease genes from PanelApp and OMIM, and identified 97,135 pathogenic and 478,263 likely pathogenic variants using an American College of Medical Genetics and Genomics-based classifier. This expanded pathogenic and likely pathogenic variants nearly six-fold. On average, an individual is born with 4.70 pathogenic or likely pathogenic variants, of which 1.66 are compatible with a Mendelian condition at the genotype level; 1 in 11 has an actionable genotype, and 382 genes are candidates for carrier screening. A genome-first approach revealed the likelihood of having a genotype compatible with disease in 13 ICD-10 disease groups, for example, congenital (1 in 2.70), musculoskeletal/connective (1 in 3.00) and blood/immune (1 in 3.07 individuals). Evidence-based genetic epidemiology demonstrates the potential of personalized medicine for the implementation of early preventive measures and incentivization of lifestyle changes to enhance healthspan and lifespan.

## Introduction

Whole-exome sequencing is a first-line diagnostic test and an integral component of research^1,2^. Genomics England launched the PanelApp to facilitate accurate gene-to-phenotype associations^3^. The Online Mendelian Inheritance in Man (OMIM, https://omim.org/) database is a curated catalog of human genes, genetic disorders, and their genotype–phenotype relationships. To standardize the clinical interpretation of genetic variations for disease genes, the American College of Medical Genetics and Genomics (ACMG) and the Association for Molecular Pathology have developed guidelines that rely on functional studies, in silico predictions, as well as population frequency and reported variations data^4^. This resulted in the designation of a variant as pathogenic (P), likely pathogenic (LP), variant of uncertain significance (VUS), likely benign (LB), or benign (B). To fully utilize this advancement, ACMG recommends clinicians implement surveillance of 84 secondary finding genes, as well as screening of all genes with a carrier frequency higher than 1 in 200^5,6^. However, variant classification from sequence data is an intricate process. To address this issue, we recently developed the Automated ACMG-based Variant Classifier (AAVC), which reaches a concordance rate of 94.4% with the Food and Drug Administration-recognized expert variant curations^7^. Additionally, for missense variants, we developed a prediction tool, FuncVEP, which achieved accuracies of 93.3% on functional assay datasets and 96.6% on ClinVar^8^.

These developments provide an unprecedented opportunity to compile a comprehensive account of the burden of inherited predisposition to disease using large-scale population data and variant prioritization methods. Indeed, several studies assessed the extent of previously reported pathogenic and high-impact variants in disease genes^9–20^. However, they fall short of evaluating the entire reported allelic diversity present in large datasets in diverse populations. Also, they often disregard the disease mechanism, functional studies, and ACMG classification. Therefore, we classified all variants in the Genome Aggregation Database (gnomAD; 730,947 individuals), Regeneron Genetics Center Million Exome (RGC-ME) (983,578 individuals) and Turkish Variome (TrVar; 3362 individuals), across nine genetic ancestry groups, using AAVC and FuncVEP, and calculated the per-individual burden of disease-associated variants, carrier frequency (CrF), and genetic prevalence (GP) for each known disease gene^14,21,22^. In addition, we resorted to the International Classification of Diseases, Tenth Revision (ICD-10), and calculated GP according to disease groups.

## Results

### Variant classification

**Fig. 1:**
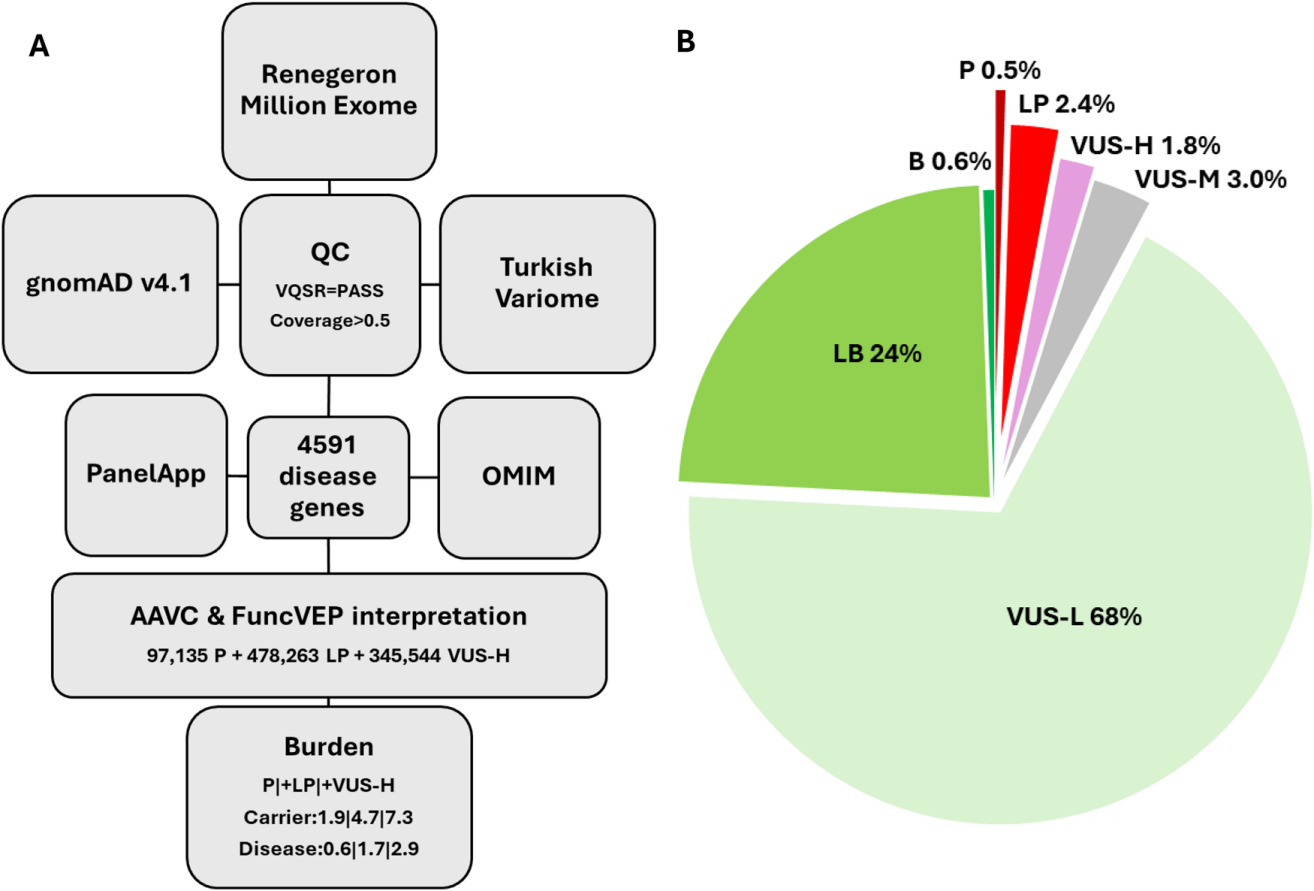
Variant classification and distribution. **A** High-quality variants were selected from gnomAD, RGC-ME and TrVar that passed the VQSR filter and covered more than half of the individuals. Variants of genes with a high level of evidence for disease association and therefore labeled “green” in PanelApp and disease gene in OMIM were selected. We used AAVC to classify variants according to ACMG guidelines. We next calculated the burden of each genetic disease based on the allele frequency information of P+LP+VUS-H variants. **B** Distribution of classified variants. P+LP+VUS-H variants, corresponding to 4.7%, were prioritized for downstream burden analyses. This figure is generated using Microsoft Office.

The PanelApp data lists 4112 disease genes^3^. However, 92 were either non-protein-coding gene or had either undefined or mitochondrial inheritance patterns. Therefore, we excluded these 92 genes and selected 4030 with well-defined autosomal dominant (AD), autosomal recessive (AR), and X-linked (XL) phenotypes for downstream analyses (Supplementary Data 1). Likewise, OMIM lists 4358 disease genes, of which 4247 are protein-coding with defined inheritance patterns and disease mechanisms (Supplementary Data 2). To capture genes from both resources, we combined the curated PanelApp and OMIM gene sets, yielding a final list of 4591 disease genes (Supplementary Data 3). Next, we subjected the variants of the 4591 genes from the gnomAD v.4.1.0, RGC-ME and TrVar to quality control steps followed by AAVC and FuncVEP classification. This yielded 97,135 P (0.5%), 478,263 LP (2.4%), 345,544 VUS-high (VUS-H) (1.8%), 591,857 VUS-mid (VUS-M) (3.0%), 13,308,271 VUS-low (VUS-L) (68.1%), 4,618,155 LB (23.6%) and 109,620 B (0.6%) variants (Fig. 1A, B, Supplementary Data 4 and Supplementary Fig. 1). ClinVar submissions corresponded to 986,458 variants (5.0%). Functional studies reported in the literature covered 17,866 (0.09%) variants (Supplementary Information: ACMG Variant Classification Guideline; Supplementary Data 5).

### Carrier frequency and genetic prevalence

**Fig. 2:**
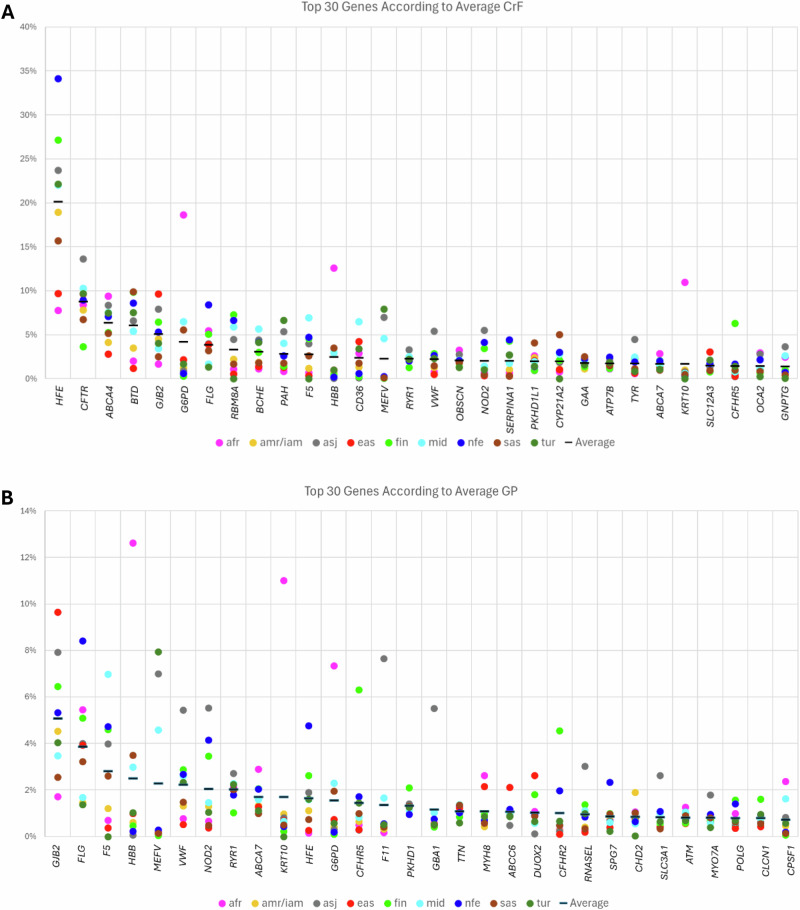
Top 30 genes according to CrF and GP. P+LP variants were prioritized across nine genetic ancestry groups. The x-axes show the gene symbols and the y-axes show the (**A**) CrF and **B** GP. The ancestries are afr (African), amr/iam (Admixed/Indigenous American), asj (Ashkenazi Jewish), eas (East Asian), fin (Finnish), mid (Middle Easterner), nfe (non-Finnish European), sas (South Asian) and tur (Turkish). This figure is generated using Microsoft Office.

We calculated the cumulative allele frequency (CAF) per gene for three variant sets—P, P+LP, and P+LP+VUS-H—in all three cohorts (Supplementary Information: VUS Subclasses; Supplementary Data 6). Because gnomAD and RGC-ME include similar ancestry groups, we assessed their concordance and observed high correlations across ancestries (mean correlation coefficients: *P* = 0.96, P + LP = 0.92, P + LP + VUS-H = 0.91), with relatively lower values for Africans (*P* = 0.99, P + LP = 0.80, P + LP + VUS-H = 0.80) and East Asians (*P* = 0.79, P + LP = 0.81, P + LP + VUS-H = 0.83) (Supplementary Data 6). For data sharing, we averaged CAF estimates between gnomAD and RGC-ME within each shared ancestry, and subsequently calculated CrF and GP for nine ancestries in total: eight shared ancestries from gnomAD and RGC-ME, plus Turkish individuals from TrVar. The top genes with the highest CrF for P+LP variants were *G6PD* (XL, favism) for Africans (1/5 individuals; 244 million individuals); and *HFE* (AR, hemochromatosis) for non-Finnish Europeans (1/3; 331 million), Finnish (1/4; 1.51 million), Ashkenazi Jews (1/4; 3.72 million), Turkish (1/5; 19 million), Middle Easterners (1/5; 110 million), Admixed/Indigenous Americans (1/5; 151 million), South Asians (1/6; 307 million) and East Asians (1/10; 184 million), which was driven by two missense variants with penetrance as low as 4.5%^23^ (Fig. 2A and Supplementary Data 7). We investigated the correlation between reported CrF and calculated CrF for a given autosomal recessive disease, and they were better correlated for non-Finnish Europeans (*R* = 0.57, *p* < 0.001) compared to non-Europeans (*R* = 0.37, *p* = 0.075), probably due to the underrepresentation of non-Europeans in epidemiological studies (Supplementary Information: Calculated and Reported Carrier Frequencies and Fig. S2; Supplementary Data 8). To further investigate this discrepancy, we conducted a case study on one of the best-documented Mendelian genes, *CFTR* with a 1 in 25 carriership for Europeans, using the *CFTR*-France database^24,25^. We calculated that 1 in 21 individuals carries a hypomorphic and 1 in 27 cystic fibrosis-causing variants, translating to an overall carriership rate of 1 in 12, suggesting hypomorphic alleles as a contributor to the discrepancy (Supplementary Information: *CFTR* variants; Supplementary Data 9).

When the genes were ordered based on GP, the most frequently observed were *HBB* (AD, hemoglobinopathies) for Africans (1/8; 165 million) and South Asians (1/29; 68 million); *GJB2* (AD, palmoplantar keratoderma) for East Asians (1/10; 183 million), Ashkenazi Jews (1/13; 1.24 million), Finnish (1/16; 0.36 million) and Admixed/Indigenous Americans (1/22; 33 million), *F5* (AD, thrombophilia) for Middle Easterners (1/14; 35 million), *FLG* (AD, dermatitis) for non-Finnish Europeans (1/12; 82 million), *MEFV* (AD/AR, familial Mediterranean fever) for Turkish (1/13; 7 million).

GP and CrF data for all disease genes can be explored using the interactive table in Supplementary Data 10 for all genetic ancestry groups.

**Fig. 3:**
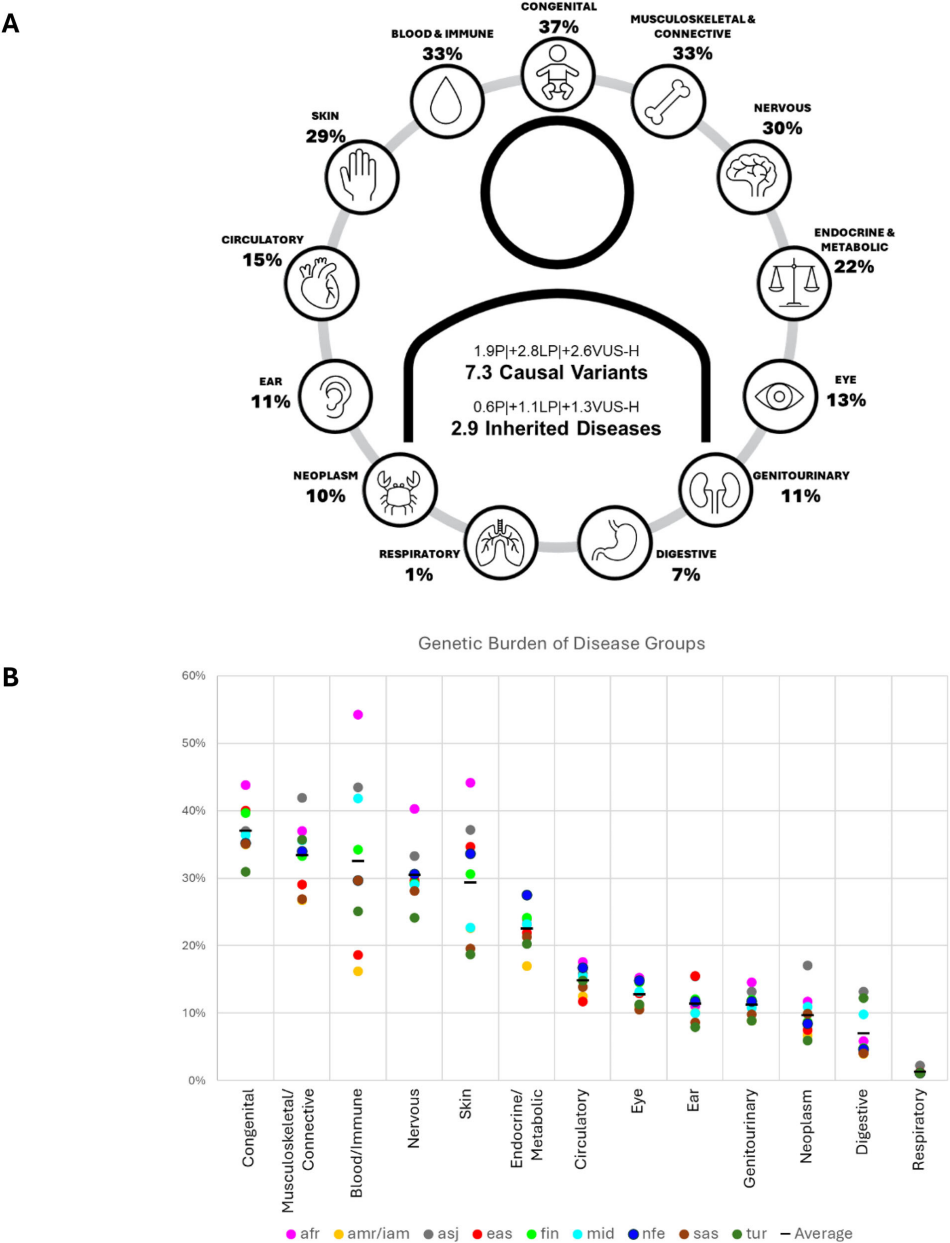
Genomic burden of inherited predisposition to diseases. **A** P+LP+VUS-H variants per individual for 4591 disease genes, and GP of P+LP variants according to ICD-10-based disease groups on average for the nine genetic ancestries. **B** GP distribution for P+LP variants according to ICD-10-based disease groups for all genetic ancestries. The x-axis shows the 13 different disease groups, and the y-axis shows the GP. The ancestries are afr (African), amr/iam (Admixed/Indigenous American), asj (Ashkenazi Jewish), eas (East Asian), fin (Finnish), mid (Middle Easterner), nfe (non-Finnish European), sas (South Asian) and tur (Turkish). This figure is generated using Microsoft Office.

Next, we calculated the expected number of variants per individual carrying any P, LP, or VUS-H variant based on CrF. The mean across all genetic ancestries was 1.90 for P, 4.70 for P+LP, 7.33 for P+LP +VUS-H. Regarding the cumulative GP of the genes, an individual had, on average, 0.60 P, 1.66 P + LP, and 2.88 P + LP + VUS-H genotype compatible with a heterozygous dominant, homozygous recessive, or hemizygous X-linked disorder for 4591 disease genes. The number changes to 1.47 and 1.58 for P+LP variants when only the PanelApp or OMIM genes are considered, respectively. The probability for an individual not to carry any P+LP variant was 1 in 107 (0.94%; 75 million) and not to carry any P+LP+VUS-H variant was 1 in 1488 (0.7‰; 5 million) (Fig. 3A, Supplementary Data 11). When considering the three cohorts separately—gnomAD, RGC-ME, and TrVar—the average cumulative CrFs for P+LP variants were 4.53, 4.94, and 4.41, and the corresponding average cumulative GPs were 1.58, 1.80, and 1.35, respectively. When restricting the analysis to ClinVar P/LP variants, the cumulative CrF was 2.02 and the cumulative GP was 0.48.

### Inherited burden according to disease groups

We adopted a genome-first approach to investigate the inherited burden for 13 disease groups compiled from ICD-10 codes and employed a bipartite matching approach between genes and phenotypes. Of the 4591 disease genes, 1859 (40%) were in a single disease group, 1467 (32%) in two disease groups, and the remaining 1265 (28%) in more than two disease groups, with the highest being *KMT2D* (Kabuki syndrome), in 9 disease groups. Next, we assessed the burden of each disease group by summing the GP of genes using P+LP variants across nine genetic ancestries. Because many genes map to multiple phenotypic categories, ICD-10 disease-group genotype prevalences are *non-mutually exclusive*, therefore an individual gene may contribute to more than one group. The average ranged from 1 in 3 to 1 in 74: congenital (37.0%; 2.82 billion), musculoskeletal/connective (33.4%; 2.30 billion), blood/immune (32.6%; 2.29 billion), nervous (30.4%; 2.33 billion), skin (29.3%; 2.25 billion), endocrine/metabolic (22.5%; 1.67 billion), circulatory (14.9%; 1.07 billion), eye (12.8%; 955 million), ear (11.4%; 858 million), genitourinary (11.2%; 839 million), neoplasm (9.7%; 686 million), digestive (7.0%; 371 million) and respiratory (1.3%; 92 million) (Fig. 3A, B, Supplementary Data 11).

The top 3 genes according to CrF in 13 disease groups and their associated disorders are listed in Table 1. When we assessed the genetic burden in each genetic ancestry separately, the likelihood of having a genetic disease was highest for blood/immune in Africans (54.3%; 710 million), Ashkenazi Jews (43.5%; 7 million), Middle Easterners (41.8%; 209 million), congenital in East Asians (40.1%; 762 million), Finnish (39.7%; 2 million), non-Finnish Europeans (35.2%; 341 million), South Asians (35.1%; 685 million), Admixed/Indigenous Americans (35.0%; 255 million) and musculoskeletal/connective in Turkish (37.7%; 31 million). The lowest burden for all genetic ancestries was for the respiratory disease group. When we adjusted the burden for dominant disorders in each disease group for all genetic ancestries, skin and blood/immune diseases ranked the highest, and nervous, congenital, respiratory, eye and neoplasm diseases ranked the lowest. A lower prevalence of P or LP variants per dominant gene suggested haploinsufficiency, which was also observed as a negative selection against the high-impact variants for nervous, neoplasm disease genes, as supported by gene constraint metrics. Moreover, genes that were classified under more than one disease group were under a stronger selection for high-impact and missense variants compared to those in only a single disease group (Supplementary Information: Gene Constraint and Fig. S3; Supplementary Data 11 and 12).

**Table 1:** The top three genes of ICD-10-based disease groups

Disease Group	Gene	I^a^	OMIM	CrF (1/x)^b^	GP (1/x)^b^
Congenital	*RBM8A*	AR	Thrombocytopenia-absent radius syndrome (274000)	−|30|30	−|2.1K^c^|2.1K
	*DHCR7*	AR	Smith-Lemli-Opitz syndrome (270400)	100|87|80	24K|20K|18K
	*MYH8*	AD	Trismus-pseudocamptodactyly syndrome (158300)	219K|92|72	219K|92|72
Musculoskeletal/Connective	*OBSCN*	AR	Rhabdomyolysis, 1 (620235)	666|48|45	1.3M^c^|8.5K|7.4K
	*RYR1*	AD	Malignant hyperthermia susceptibility 1 (145600)	531|49|32	531|49|32
	*TTN*	AR	Muscular dystrophy, limb-girdle, 10 (608807)	330|92|52	412K|32K|10K
Blood/Immune	*G6PD*	XLR	G6PD deficiency (300908)	35|24|24	93|65|64
	*F5*	AD/AR	Thrombophilia (188055)	36|36|35	36|36|35
	*HBB*	AD	Delta-beta thalassemia (141749)	42|40|34	42|40|34
Nervous	*ABCA7*	AD	Alzheimer disease 9 (608907)	6K|59|55	6K|59|55
	*SMPD4*	AR	Neurodevelopmental disorder (618622)	9.2K|115|112	147M|37K|35K
	*SPG7*	AD/AR	Spastic paraplegia 7 (607259)	139|116|93	139|116|93
Skin	*FLG*	AD	Dermatitis, atopic, susceptibility to, 2(605803) (AD)	48|26|20	48|26|20
	*TYR*	AR	Albinism, oculocutaneous, type IB (606952)	68|58|51	11K|8.8K|7.8K
	*KRT10*	AD	Epidermolytic hyperkeratosis 2A (620150)	112K|59|58	112K|59|58
Endocrine/Metabolic	*HFE*	AR	Hemochromatosis (235200)	5|5|5	61|61|61
	*BTD*	AR	Biotinidase deficiency (253260)	17|16|16	842|787|761
	*PAH*	AR	Phenylketonuria (261600)	38|35|34	3.6K|3.2K|3.1K
Circulatory	*MYH7*	AD	Congenital myopathy 7A (608358)	1.1K|189|131	1.1K|189|131
	*LPA*	AD	Coronary artery disease, susceptibility to (618807)	−|229|60	−|229|60
	*SLC4A3*	AD	Short QT syndrome 7 (620231)	10M|263|198	10M|263|198
Eye	*ABCA4*	AD/AR	Macular degeneration, 2(153800),	28|16|15	2.6K|827|744
	*USH2A*	AR	Retinitis pigmentosa 39 (613809)	126|76|51	54K|21K|9.2K
	*EYS*	AR	Retinitis pigmentosa 25 (602772)	160|77|62	59K|19K|13K
Ear	*GJB2*	AD/AR	Deafness 3A (601544)	21|20|19	21|20|19
	*PKHD1L1*	AR	Deafness 124 (620794)	257|50|46	103K|8K|6.9K
	*SLC26A4*	AR	Deafness 4, with enlarged vestibular aqueduct (600791)	97|77|69	34K|22K|18K
Genitourinary	*CYP21A2*	AR	Congenital adrenal hyperplasia (201910)	152|50|41	55K|6.5K|4.9K
	*SLC12A3*	AR	Gitelman syndrome (263800)	111|66|50	43K|14K|8.3K
	*PKHD1*	AR	Polycystic kidney disease 4 (263200)	230|76|62	117K|22K|15K
Neoplasm^d^	*RNASEL*	AD	Prostate cancer 1 (601518)	−|105|88	−|105|88
	*ATM*	AD	Breast cancer (114480)	321|122|100	321|122|100
	*CHEK2*	AD	Tumor predisposition syndrome 4 (609265)	344|159|88	344|159|88
Digestive	*ATP7B*	AR	Wilson disease (277900)	81|57|48	23K|12K|8.6K
	*ALDOB*	AR	Fructose intolerance (229600)	145|107|87	63K|39K|28K
	*UGT1A1*	AR	Crigler-Najjar syndrome (218800;606785)	1K|147|106	2.2M|52K|31K
Respiratory	*CFTR*	AR	Cystic fibrosis (219700)	30|11|10	3.2K|429|308
	*SERPINA1*	AR	Emphysema due to AAT deficiency (613490)	182|49|20	72K|5.9K|963
	*DAW1*	AR	Ciliary dyskinesia, primary, 52 (620570)	6.7K|84|70	52M|20K|16K

^a^Inheritance.

^b^CrF and GP in the order of P|P+LP|P+LP+VUS-H variants.

^c^K, thousand, M, million.

^d^The neoplasm disease group selects genes based on Genetic Prevalence instead of Carrier Frequency.

### Secondary finding genes

ACMG secondary findings v3.3 lists 84 genes as actionable and recommends reporting all P + LP variants for 83 of them plus only the C282Y variant for *HFE*^6^. When SNPs and short indels of the 84 ACMG secondary finding genes were analyzed, cumulative CrF for P+LP variants across genetic ancestries ranged from 16.5% for East Asians to 36.3% for non-Finnish Europeans. The highest CrF after averaging across nine genetic ancestries was for *BTD* (AR; biotinidase deficiency; 1/16; 388 million), followed by *HFE (*AR; hemochromatosis; 1/27; 208 million) and *RYR1* (AD; Malignant hyperthermia; 1/49; 151 million) (Supplementary Data 11 and 13).

Next, we investigated the cumulative GP, which ranged from 6.8% (Finnish) to 11.5% (African) for the 84 genes. The highest GP was observed for *RYR1* (AD; Malignant hyperthermia; 1/49; 151 million), *TTN* (AD; dilated cardiomyopathy; 1/92; 92 million), and *LDLR* (AD; familial hypercholesterolemia; 1/181; 47 million) across nine genetic ancestries. With respect to disease groups as defined by the ACMG Secondary Findings Working Group, the burden was highest for cardiovascular (1/21; 368 million) followed by miscellaneous (1/37; 216 million), cancer (1/78; 88 million) and inborn errors of metabolism (1/333; 23 million) phenotypes (Supplementary Data 11 and 13)^6^.

### Candidate genes for screening

ACMG recommends screening for genetic disorders with a CrF higher than 1 in 200 for all couples^5^. The current list contains 286 genes selected based on reported P+LP variants^5,18,26^. When we used ACMG guidelines for variant classification, 96 additional genes became candidates for screening, thus increasing the number to 382. Different sets of genes met the ACMG screening criteria across the nine genetic ancestries, and 22 genes were shared by all of them (Supplementary Information: Carrier Screening; Supplementary Data 14).

## Discussion

The scope of inherited burden for disease predisposition in diverse populations is an open question. ACMG guidelines for variant classification, combined with large population variation databases, promise to provide valuable answers, especially using a genotype-first approach. By contrast, phenotype-first prevalence estimates are constrained by incomplete national health records and imperfect case ascertainment. Moreover, misdiagnosis represents an obstacle to frequency calculations, primarily for diseases such as Wilson disease, with a correct diagnostic rate as low as 7%^27^. This is especially the case for determining the true CrF and GP of rare disorders. Therefore, the gene-centric approach presented here provides a unique perspective on the inherited burden for P+LP variants in 4591 known disease-causing genes across nine genetic ancestries. After standardizing the variant classification process by using AAVC, we observed that an individual carries, on average, 4.70 P+LP variants, of which 1.66 are compatible with a heterozygous dominant, homozygous recessive, or hemizygous X-linked disorder, 1 in 11 individuals carry an actionable genotype, and 382 genes are candidates for carrier screening.

The effects of heterozygote advantage and population bottleneck were observed as CrF differences across different genetic ancestries. For example, high CrF of *HBB* and *G6PD* in Africans can be attributed to malaria resistance. Similarly, high frequency of *MEFV* variants in Turkish, Middle Easterners and Ashkenazi Jews have been proposed to protect against *Yersinia pestis* infections, *CFTR* and *GJB2* variants against diarrhea, and *CYP21A2* variants have been hypothesized to decrease mortality from pneumonia^28–31^. *DHCR7* variant carriers have shown to exhibit higher levels of vitamin D, which might explain its high prevalence in non-Finnish Europeans^32^. On the other hand, *ASPA*, *ELP1*, *G6PC1*, and *HEXA* in the Ashkenazi Jews and *AGA*, *BCS1L*, *CLN5*, and *SLC17A5* in the Finnish, had higher CrFs probably due to population bottlenecks^33,34^. We noted discrepancies between the reported and calculated CrFs, which probably arises from inbreeding, isolation, assortative mating, incomplete penetrance, locus heterogeneity, linkage disequilibrium, environmental factors for complex diseases, and under- or over-estimated frequencies due to false negatives or false positives^12,35^. This was further exacerbated by the underrepresentation of non-Europeans in gnomAD, and a low number of epidemiological studies for such populations, which led to less accurate estimations for CrF and a higher discordance between the reported and calculated CrF. Incomplete penetrance likely contributed to the discrepancy between GP and reported prevalence in certain AD diseases such as *TGIF1-*associated holoprosencephaly, or hypomorphic variants in AR phenotypes like cystic fibrosis or hemochromatosis, particularly when the least conservative variant set (P+LP+VUS-H) was used^36^.

When the burden was assessed according to ICD-10-based groups, the highest was for congenital followed by musculoskeletal/connective, blood/immune, nervous, and skin diseases when all genetic ancestries were considered. By extending variant detection beyond short-read single-nucleotide and small indel variants to include large insertions/deletions, copy-number variants, pathogenic repeat expansions, and epigenetic alterations (e.g., methylation-mediated gene silencing), which underlie disorders such as Duchenne muscular dystrophy, α-thalassemia, spinal muscular atrophy, and Huntington disease, future long-read genome sequencing studies are expected to further increase and refine our estimates of inherited burden. In parallel, large-scale efforts such as the Impact of Genomic Variation on Function (IGVF) Consortium will improve the classification of non-coding, regulatory, and missense/in-frame indel variants, leading to progressively more accurate prevalence and carrier-frequency estimates than those reported here^37^.

We anticipate that our findings will serve as a valuable resource for epidemiological studies as well as decision-making processes regarding the screening, diagnosis, and treatment of inherited diseases. From an individual’s perspective, one can learn about their genetic risk and, in some cases, have an opportunity to take preventive measures or initiate lifestyle modifications. We estimated an actionable-genotype prevalence of 1 in 11 individuals (1.71 billion globally) with at least one reportable P/LP actionable genotype across the 84 ACMG-recommended secondary finding genes, with potential implications for healthspan and lifespan. For example, a recent study demonstrated that the presence of an actionable genotype in a cancer-related gene was associated with a 3-year reduction in survival compared to noncarriers, and most deaths among carriers were attributed to cancer-related conditions^20^. Previous studies have reported 1.1–1.58 P/LP variants per individual, with 1 in 25 to 1 in 37 individuals carrying an actionable genotype, compared with our estimates of 4.70 variants per individual and 1 in 11 carrying an actionable genotype^9,14,20^. This discrepancy may reflect reliance on previously reported or high-impact variants rather than on systematic ACMG-based variant classification and determination of disease mechanisms. In addition, reported variants are likely to be biased toward well-studied populations, leading to underrepresentation of understudied groups^38^. From a health policy perspective, frequency estimates of certain genetic diseases can facilitate health economics analysis. Our findings can be utilized to identify candidate genes for population-specific genetic screening tests, which would benefit the equitable carrier screening of parents or newborns. As the cost of sequencing decreases, it can be foreseen that sequencing every newborn at birth will soon be feasible. A gene-centric approach that incorporates reverse phenotyping in a clinical setting promises to improve diagnostic accuracy and, thus, precision medicine interventions significantly^39^. Lastly, from a societal standpoint, we hope this research will decrease discrimination and social stigmatization associated with inherited diseases, as an overwhelming majority of individuals are expected to carry germ-line risk variants on average.

## Methods

### Selection of disease gene list

The Genomics England PanelApp gene list was downloaded using the API on November 24, 2025^3^. PanelApp prioritizes genes with a Mendelian pattern of causation appropriate for reporting in a diagnostic setting, with the exclusion of intermediate and low penetrance genes. The “green” genes, indicating high-level evidence of disease-genes association, were prioritized, yielding 4112 disease genes across 416 panels. Of the 4112 genes 4071 were protein-coding genes, and non-protein-coding genes were excluded from analysis, since they were not thoroughly covered by exome sequencing. The breakdown of the inheritance for the 4071 genes was 2147 autosomal recessive, 1134 autosomal dominant, 525 both autosomal dominant and recessive, 221 X-linked, 13 mitochondrial, two pseudoautosomal recessive and one pseudoautosomal dominant.

Inheritance was not ascertained for 28 genes. We retained 4030 of 4071 protein-coding PanelApp genes for downstream analyses, excluding 41 genes with unascertained/undefined (*n* = 28) or mitochondrial (*n* = 13) inheritance patterns (Supplementary Data 1).

Disease-gene associations from OMIM were downloaded on November 24, 2025. There were 4358 genes associated with 6173 phenotypes. Of the 4358 genes 4333 were protein-coding genes. There were 2296 autosomal recessive, 1209 autosomal dominant, 589 both autosomal dominant and recessive, 230 X-linked, 5 digenic, one Y-linked, two pseudoautosomal recessive and one pseudoautosomal dominant. Short variants of the genes leading to disease by only repeat expansion were also removed since such variations were absent from the exome data. Additionally, genes without established disease mechanism (loss of function, gain of function, neomorphic variation etc.) were also excluded. We subjected remaining 4247 genes for downstream analyses (Supplementary Data 2).

For the final disease gene list we combined both PanelApp and OMIM lists resulting in 4591 disease genes, while 3686 genes were overlapping for both lists, 344 genes were unique to PanelApp list and 561 genes were unique to OMIM list (Supplementary Data 3).

### Determination of disease mechanism

The disease mechanism of a gene can be loss of function (LoF), gain of function, including the neomorphic function (GoF), or LoF+GoF. LoF+GoF genes could lead to a similar phenotype in both LoF and GoF direction, such as *STAT1*; or a new one, such as *ANO5*, which leads to LoF (Miyoshi muscular dystrophy) or GoF (Gnathodiaphyseal dysplasia)^40,41^. To curate a list of GoF and LoF + GoF disease genes, we parsed OMIM in November 2025 for “gain of function” OR “gain-of-function” OR “toxic gain of function” OR neomorph* prompts. This yielded 807 genes and they were given a preliminary GoF gene status. Each of the 807 genes was manually reviewed and curated to determine the disease mechanism. This resulted in 4292 LoF, 241 GoF, 58 LoF + GoF genes. Twenty-one out of 58 LoF + GoF genes had dominant GoF and recessive LoF phenotype. This gene-phenotype association level categorization necessarily simplifies a broader spectrum of directional effects. As recommended by ACMG, LoF-associated scores provided by AAVC, PVS1, were removed for GoF gene-phenotype pairs. Although end-truncating variants can lead to a GoF effect, *NOTCH2* related skeletal disorders, there is no established guideline for the detection of such cases^42^ (Supplementary Data 3). Additionally, emerging tools that predict the functional direction of variants, particularly missense and splicing variants, together with updated ACMG guidelines, are expected to further refine the classification of potentially disease-causing variants according to their underlying mechanism.

### Distribution of genes to disease groups

We next distributed the panels and OMIM phenotypes into 13 disease categories based on the ICD-10 grouping system, allowing for the inclusion of a single gene in multiple disease groups. Ten panels in PanelApp were for general screening or test for drug responses and therefore did not bin into a disease group. The number of ICD-10 disease groups assigned for a gene ranged from nine for a single gene, *KMT2D*, to one for 1859 genes. For example, *CFTR* was assigned to five different disease groups: blood/immune, congenital, digestive, endocrine/metabolic, and respiratory (Supplementary Data 3).

### Variant selection, annotation, and classification

gnomAD exome v4.1.0 variant call format (VCF) files containing 730,947 exomes were downloaded on May 23, 2024 (https://gnomad.broadinstitute.org/data#v4-variants). The dataset includes summary statistics for the following genetic ancestry groups: African/African American (afr), Admixed American (amr), Ashkenazi Jewish (asj), East Asian (eas), Finnish (fin), Middle Eastern (mid), non-Finnish European (nfe), and South Asian (sas). RGC-ME data were downloaded on June 26, 2025 (https://rgc-research.regeneron.com/me/home)^14^. The VCF file contained data from 983,578 individuals across eight genetic ancestries: African, Ashkenazi Jewish, East Asian, European, Finnish, Indigenous American (iam), Middle Eastern, and South Asian. To harmonize the RGC-ME ancestries with those in gnomAD, we defined a non-Finnish European group by removing Finnish, Ashkenazi Jewish and Amish (ami) individuals from the EUR ancestry. The Turkish Variome dataset contains summary statistics generated from 3362 Turkish individuals (tur), who are genetically distinct from both Middle Eastern and European populations^22^. All three datasets provide allele count (AC) and allele number (AN) information stratified by genetic ancestry.

We selected high-quality variants that passed the variant quality score recalibration (VQSR) filter (PASS) and covered more than 50% of individuals for corresponding ancestries for 4591 disease genes for each cohort, leaving 13,481,404 variants in gnomAD, 14,534,227 variants in RGC-ME cohort, 367,962 variants in Turkish Variome. After the removal of overlapping values 19,548,845 unique variants for 4591 disease genes remained.

AAVC is a program that classifies germline variants in accordance with ACMG variant classification guidelines. It adopts a Bayesian framework to combine potentially conflicting lines of evidence, such as a pathogenic in silico prediction (PP3) in the presence of a common variant frequency (BS1). Based on the interpretation of 21 out of 28 ACMG criteria, AAVC classifies variants into seven broad categories of P (odds of pathogenicity >99%), LP (90%), VUS-H (67.5%), VUS-M (41%), VUS-L (14%), LB (10%), and B (<1%) (Supplementary Information: VUS Subclasses)^43^.

FuncVEP, functional-trained variant effect predictor, is a missense predictor designed to be used under the in silico prediction criteria for ACMG classification and intentionally kept independent of other criteria, such as splice prediction and allele frequency. It is trained on a diverse set of functionally validated variants and outperforms other missense predictors on both clinical and functional benchmarks, facilitating the identification of new gene–phenotype associations^8^.

Ensembl Variant Effect Predictor release 114 was used for the pre-annotation required for AAVC^44^.

AAVC was run with the --vcf_mode and --activate_PP5_BP6 options, with 500,000 variants per batch separated to 40 parallel instances. Canonical transcripts for the 4591 disease genes, based on Ensembl release 114, were selected for classification. However, AAVC can also process alternative transcripts. FuncVEP’s CTE model was used with defined PP5 and BP6 thresholds.

### Statistical analysis of carrier frequency and genetic prevalence

Allele frequency (AF) for a variant was calculated as ${AF}={AC}/{AN}$. For autosomal and pseudo-autosomal genes, CAF for a gene was calculated by adding allele frequencies of each P, P + LP, or P + LP + VUS-H variant, assuming they were independently assorted. CrF, which denotes the proportion of individuals in a population who have a single copy of a genetic variant, was calculated utilizing CAF and Hardy-Weinberg Equilibrium as Eq. 1:

1 $${CrF}=2* {CAF}* \left(1-{CAF}\right)$$

The sum of homozygote and compound heterozygote frequency (HomF) was calculated by squaring CAF. The GP of each compound heterozygote (CompHet) was calculated by using the AF of each allele as the Eq. 2:

2 $${CompHet}={\left({{AF}}_{i}+{{AF}}_{{ii}}\right)}^{2}-{{AF}}_{i}^{2}-{{AF}}_{{ii}}^{2}=2* {{AF}}_{i}* {{AF}}_{{ii}}$$

where AF_i_ and AF_ii_ are the allele frequencies of 2 different alleles.

GP, which denotes the proportion of a population that has a causal genotype for a genetic disorder, was assumed to be CrF for AD or AD/AR genes and HomF for AR genes. For X-linked recessive genes, we calculated GP as Eq. 3:

3 $${CAF}* ({CAF}+1))/2$$

and for X-linked dominant genes as Eq. 4:

4 $$\left(1-{CAF}\right)* {CAF}+\frac{{CAF}* \left({CAF}+1\right)}{2}$$

The likelihood of an individual not carrying any variant was calculated with CrF of all genes as Eq. 5:

5 $${Non}-{Carrier}\,{Likelihood}=\mathop{\prod }\limits_{i=1}^{n}\left(1-{{CrF}}_{i}\right)$$

where *n* is the number of all 4591 disease genes.

For cumulative GP of ICD-10 disease groups, each gene was counted once for each inheritance category they have; for example, *ANO5*, both dominant, gnathodiaphyseal dysplasia (GoF), and recessive, muscular dystrophy (LoF) was in Musculoskeletal/Connective group. Thus, the GP for both phenotypes was summed, while the cumulative GP in the Musculoskeletal/Connective group was calculated. A similar approach was adopted to calculate the cumulative GP for all genes, and each gene was counted once for both dominant and recessive phenotypes, while for cumulative CrF, each gene was counted once regardless of inheritance.

To test the correlation between reported CrF (rCrF) and estimated CrF for autosomal recessive diseases, we used a linear regression model for log-transformed values. rCrF is calculated based on the reported prevalence (rP) of a given Mendelian disease using the following Eq. 6:

6 $${rCrF}=2* \sqrt{{rP}}* \left(1-\sqrt{{rP}}\right)$$

For the list of all curated rCrF from the literature, please see Supplementary Data 8. Population sizes for each genetic ancestry group are provided in Supplementary Data 15 (World Bank data 2023 and other linked sources where appropriate); their sum (*n* = 7.47 billion) was used as the reference population for converting prevalences to absolute counts.

### Gene Constraint Metrics

pLI, LOEUF, and missense and synonymous Z scores were downloaded from https://gnomad.broadinstitute.org/data#v4-constraint for gnomAD version 4^21^. Genes were deemed constrained for different metrics with different thresholds, LOEUF < 0.6, Missense *Z* > 3.09, Synonymous *Z* > 3.71^14,45,46^. Although the original recommendation for LOEUF was <0.35, it was updated to <0.6 for gnomAD version 4 (https://gnomad.broadinstitute.org/news/2024-03-gnomad-v4-0-gene-constraint/#loeuf-guidance) (Supplementary Information: Gene Constraint).

## Supplementary information

Supplementary Information.

Supplementary Data 1.

Supplementary Data 2.

Supplementary Data 3.

Supplementary Data 4.

Supplementary Data 5.

Supplementary Data 6.

Supplementary Data 7.

Supplementary Data 8.

Supplementary Data 9.

Supplementary Data 10.

Supplementary Data 11.

Supplementary Data 12.

Supplementary Data 13.

Supplementary Data 14.

Supplementary Data 15.

## Data Availability

gnomAD v4.1.0 allele frequency data is available at https://gnomad.broadinstitute.org/data. RGC-ME data can be accessed at https://rgc-research.regeneron.com/me/home. TrVar can be downloaded from referenced publication. AAVC is freely available at for single query searches https://aavc.bilkent.edu.tr/ and https://github.com/OzcelikLab/AAVC for local runs. All data generated during this study are included in the supplementary data and files; raw data for variant classifications is available at https://zenodo.org/records/17907312 and GP and CrF data for all disease genes can be explored using the interactive table in Supplementary Data 10 for all genetic ancestry groups.
