# Supplementary material for: Inherited burden for disease predisposition in diverse populations
Source: NPJ Genom Med. 2026 Feb 18;11:18. doi: 10.1038/s41525-026-00552-5 (PMC13031552; doi:10.1038/s41525-026-00552-5)
Supplement: Supplementary file 1 — Supplementary Information. [file 41525_2026_552_MOESM1_ESM.pdf]

Inherited burden for disease predisposition in diverse populations

Barış Kayaalp, Meltem Ece Kars, Yuval Itan, Ayşe Nazlı Başak, Jean-Laurent Casanova, and Tayfun Özçelik

Supplementary Notes

ACMG Variant Classification Guideline

AAVC is an automated framework that implements 21 of the 28 ACMG/AMP criteria using current ClinGen Sequence Variant Interpretation and disease-specific VCEP specifications for germline variant interpretation[1,2]. It programmatically aggregates population frequencies, clinical assertions, regional and gene-level constraint metrics, in silico prediction scores, multiplexed assays of variant effect, text-mined functional evidence from ClinVar submissions, curated protein features, and splicing resources to evaluate each applicable ACMG evidence code. Evidence is combined with the Bayesian points-based scheme of Tavtigian et al. to derive posterior probabilities of pathogenicity and assign each variant to a 5-tier ACMG class (benign, likely benign, VUS, likely pathogenic, pathogenic), with additional subclassification of VUS into VUS-high, VUS-mid, and VUS-low to reduce residual uncertainty[3]. AAVC is available as a web-based and offline tool that accepts VCF input, supports parallel processing for large datasets.

For the popuşation dataset, the concordance of AAVC with the reported variants ranged from 99.0% for all entries to 99.5% for above 1-star entries (Supplementary Data 5).

VUS Subclasses

The rationale behind the inclusion of subclasses in VUS, low and high, was first delineated at the presentation "ACMG v4 Sequence Variant Guidelines" by Steven Harrison on September 15, 2023 (https://clinicalgenome.org/tools/clingen-summer-workshop-series-2023/sept-15-2023/), which is further supported by our previous studies on AAVC. The inclusion of subclasses increased the concordance of AAVC

with the Food and Drug Administration-approved variant list without any major sensitivity and specificity drawbacks[2].

Calculated and Reported Carrier Frequencies

The list consisted of 68 different entries after accounting for different ancestries, of which 43 were from non-Finnish European and 26 from non-European groups. Later, we removed the *BLM* gene for the Ashkenazi population since the main driver variant was the variant blm^{Ash}, a complex indel absent in gnomAD, leaving 67 entries to test.

Overall, the P+LP group provided closer CrF estimations to previously reported estimates than that of P or P+LP+VUS-H (Fig. S1 and Supplementary Data 8). For instance, the estimated CrFs of *ATP7B*, which leads to Wilson's Disease, have a reported CrF of 1 in 90 for Europeans, and our estimated CrF ranges from 1 in 76 to 1 in 37. On the contrary, for *ALDH3A2*, the cause of Sjogren-Larsson syndrome, the CrF, 1 in 1410 to 468, was insufficient to elucidate the reported prevalence rate, 1 in 251.

CFTR Variants

Our approach suggested that the carrier rate is 1.4 times to 2.9 times higher across different variant sets. Leveraging the data from *CFTR*-France September 2024, we downloaded data (https://cftr.chu-montpellier.fr/), and there were 550 disease-causing variants, 41 of which were structural variants. We converted the remaining 509 variants to genomic positions using GeneBe (https://genebe.net/tools/hgvs). The genomic position is used to match the variants with their allele frequency. Two hundred forty-six variants had allele frequency information for non-Finnish Europeans. 1 in 27 individuals carried a CF-causing variant, while 1 in 21 carried a hypomorphic variant; in total, 1 in 12 individuals carried a variant related to either CF-causing or CF-related disorder.

Allele frequency data was also used to calculate the genotype prevalence of all possible homozygous and compound heterozygous *CFTR* variant combinations (Supplementary Data 7)[4]. The most common causal genotype for cystic fibrosis or related disorder was Δ508/R668C compound heterozygote followed by Δ508 homozygote with 1 in 3313 and 1 in 4455, respectively. Assuming full penetrance for CF-causing homozygotes

and compound heterozygotes, GP was calculated as 1 in 2909, which becomes 1 in 2490 with the inclusion of variants with varying clinical consequences and CF-causing compound heterozygotes. The expected prevalence of *CFTR*-related disorder was 1 in 1219. The compound heterozygotes or homozygotes of variants that were in the unclassified group were proposed to lead to phenotype; however, given their rarity, it has not been defined yet, and their expected genetic prevalence was 1 in 80,585. 1 in 1734 individuals were either homozygous or compound heterozygous for hypomorphic *CFTR* variants, and they had no expected phenotype.

Gene Constraint

Genes associated with Mendelian diseases have been reported to be more constrained[5–7]. When compared to genes with no reported disease association in PanelApp + OMIM (LOEUF=19%, missense-z=6%, syn-z=0.06%), a higher proportion was constrained for the 4591 disease genes (LOEUF=31%, missense-z=16%, syn-z=0%), for both missense and pLoF variants. However, no such difference was observed for synonymous variants.

2472 genes out of 4591 were labeled with more than one disease group. We observed that genes that have more than one disease group assigned (LOEUF=33%, missense-z=19%, syn-z=0%) were significantly more constrained compared to genes with a single disease group (LOEUF=27%, missense-z=12%, syn-z=0%), except for synonymous z-score values. Genes associated with neoplasms were under the strongest selective pressure, followed by those linked to circulatory diseases and the nervous system (Fig. S4A and S4B, and Supplementary Data 12).

Carrier Screening

Tier 3 genetic screening for genetic disorders with a carrier frequency higher than 1 in 200 is recommended by ACMG for all couples[8]. Previous studies used various gnomAD versions, including 4.1, for the detection of candidate genes for genetic screening; however, they were mainly based on reported pathogenic variants[8–10]. Our approach revealed that 192 genes were suitable for screening for at least one genetic ancestry group when using P variants, and the number increased to 382 genes when P+LP and 570 for P+LP+VUS-H variants

were used. For the list of all genes and their recommended ancestries, please see Supplementary Data 14. The number of genes to be screened for different populations ranged from 92 to 163 for different genetic ancestries for P+LP variants (Fig. S3).

Supplementary Figures

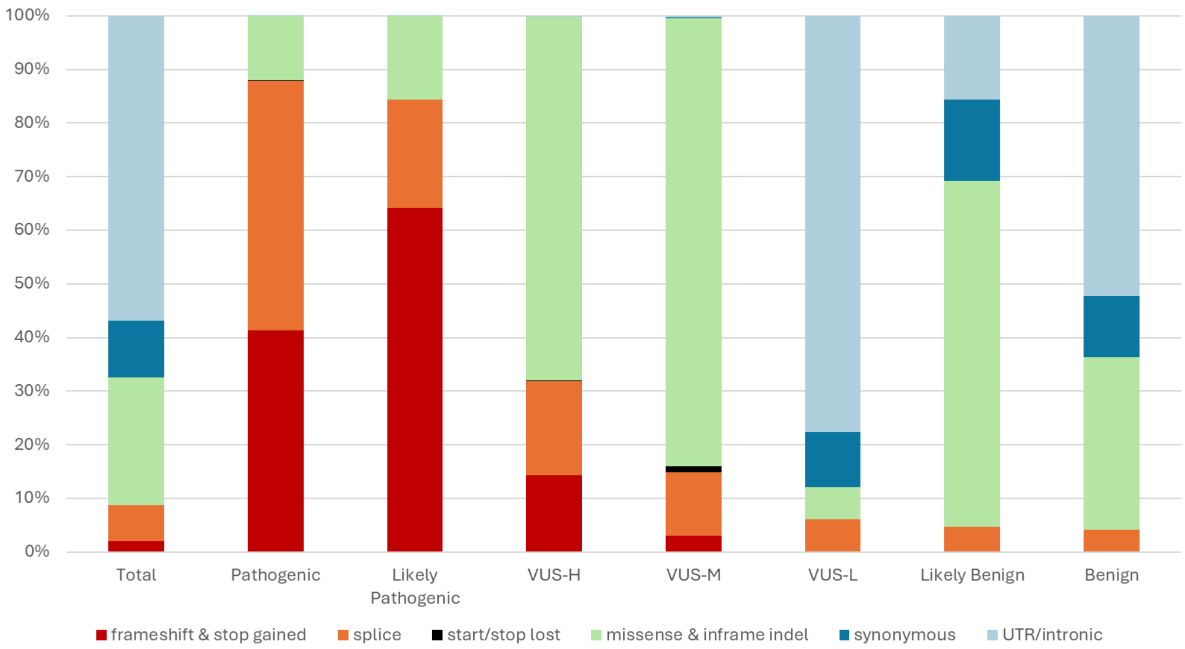

Fig. S1 Distribution of variant effects based on ACMG classification. The stacked column graph shows the distribution of variant effects based on ACMG classifications assigned by AAVC.

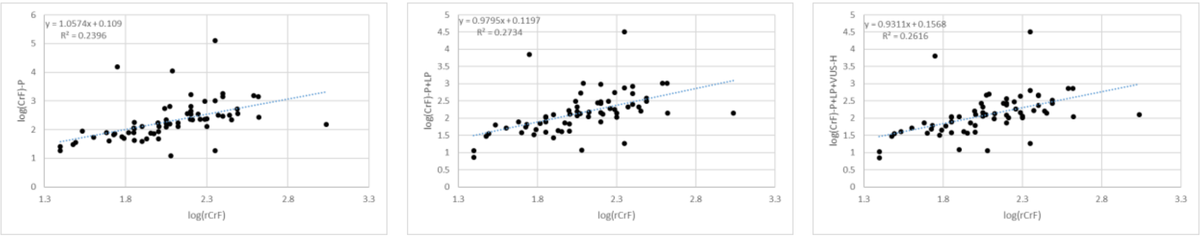

Fig. S2 Correlation of reported and estimated CrF. Correlation of reported and estimated CRF Correlation of reported and estimated prevalence for P, P+LP, and P+LP+VUS-H variants. Reported and estimated CrF were both log-transformed with a base of 10. Dots represent recessive diseases, the x-axis represents the log-transformed values of CrF, and the y-axis represents the log-transformed values of rCrF.

A.

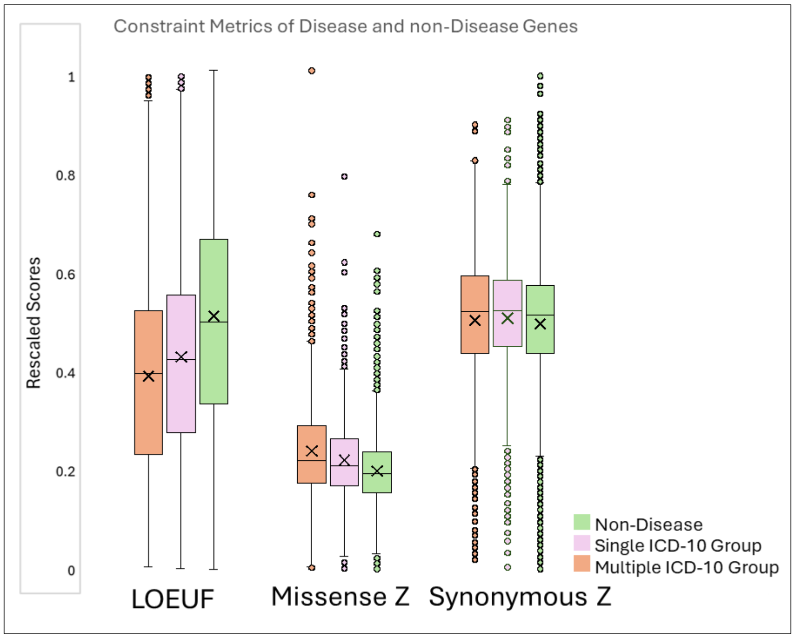

B.

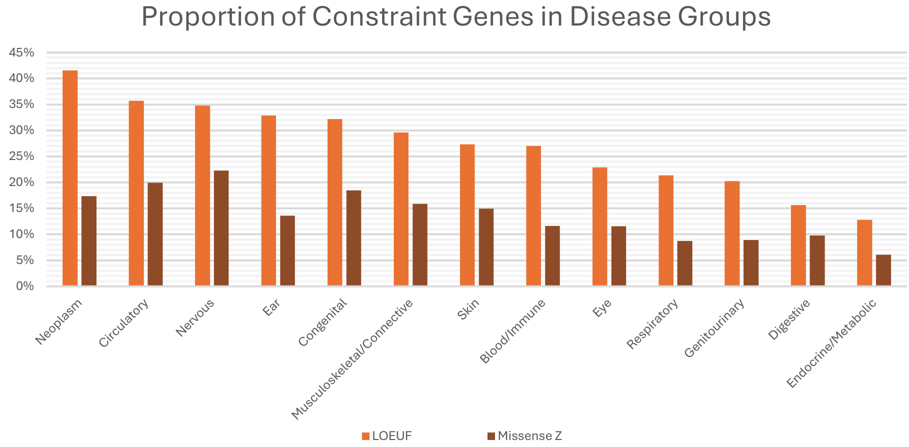

Fig. S3 Constraint of Disease Genes. **(A)** The box plot delineates the differences in constraint metrics between non-disease-associated genes, genes labeled with one disease group, and more than one disease group. Dots represent individual genes, colored boxes show the central quartiles of the distribution in each constraint metric, horizontal bars within boxes show the medians, and crosses show the means. The scores for each metric were rescaled via min-max normalization and shown on the *y*-axis. **(B)** The clustered column chart represents the proportion of genes that were classified as constrained according to different metrics on the *y*-axis and disease groups on the *x*-axis (Thresholds for constraint: LOEUF < 0.6, Missense Z-score> 3.09).

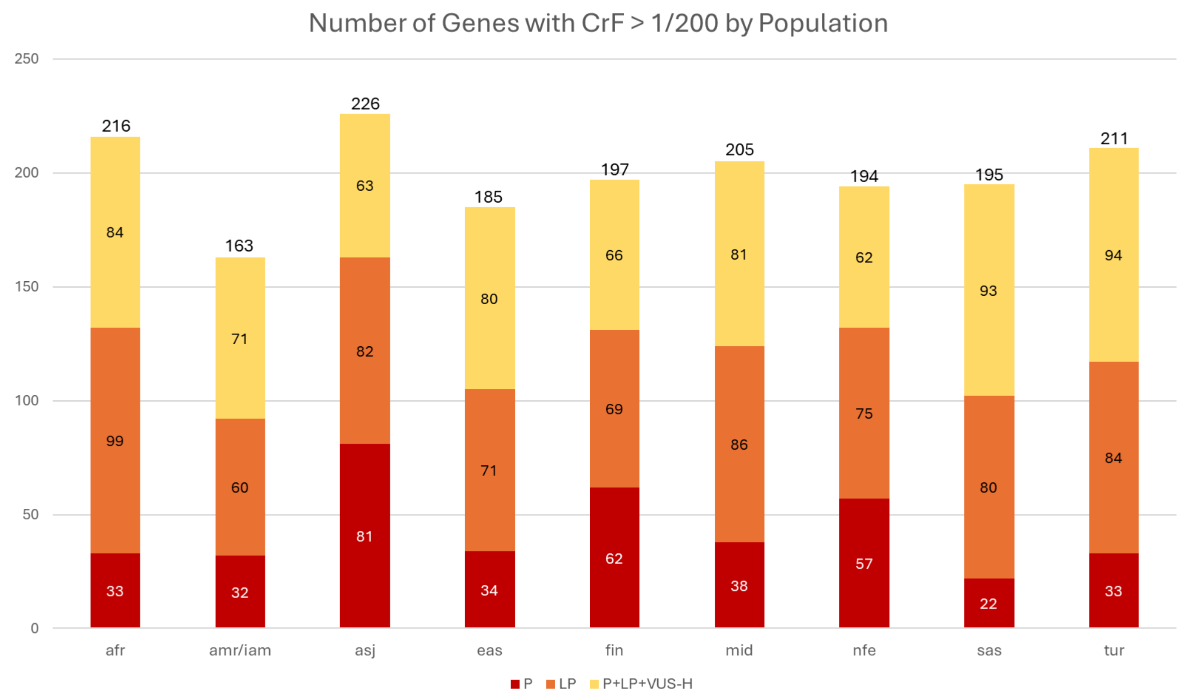

1. **Fig. S4** Candidate genes for carrier screening. The bar chart illustrates the number of candidate genes for Tier 3 carrier screening.

References

1. Richards, S. *et al.* Standards and guidelines for the interpretation of sequence variants: a joint consensus recommendation of the American College of Medical Genetics and Genomics and the Association for Molecular Pathology. *Genet Med* 17, 405–24 (2015).
2. İnan, R. A. Automated sequence variant classification tool for DNA diagnostics. (İhsan Doğramacı Bilkent University, Ankara, 2024).
3. Tavtigian, S. V., Harrison, S. M., Boucher, K. M. & Biesecker, L. G. Fitting a naturally scaled point system to the ACMG/AMP variant classification guidelines. *Hum Mutat* 41, 1734–1737 (2020).
4. Claustres, M. *et al.* CFTR-France, a national relational patient database for sharing genetic and phenotypic data associated with rare CFTR variants. *Hum Mutat* 38, 1297–1315 (2017).
5. Cassa, C. A. *et al.* Estimating the selective effects of heterozygous protein-truncating variants from human exome data. *Nature Genetics 2017 49:5* 49, 806–810 (2017).
6. Weghorn, D. *et al.* Applicability of the Mutation–Selection Balance Model to Population Genetics of Heterozygous Protein-Truncating Variants in Humans. *Mol Biol Evol* 36, 1701–1710 (2019).
7. Sun, K. Y. *et al.* A deep catalogue of protein-coding variation in 983,578 individuals. *Nature* 631, 583–592 (2024).

8. Gregg, A. R. *et al.* Screening for autosomal recessive and X-linked conditions during pregnancy and preconception: a practice resource of the American College of Medical Genetics and Genomics (ACMG). *Genetics in Medicine* 23, 1793–1806 (2021).

9. Schmitz, M. J. *et al.* Carrier frequency of autosomal recessive genetic conditions in diverse populations: Lessons learned from the genome aggregation database. *Clin Genet* 102, 87–97 (2022).

10. Schmitz, M. J. *et al.* Leveraging diverse genomic data to guide equitable carrier screening: Insights from gnomAD v.4.1.0. *The American Journal of Human Genetics* 0, (2024).
